# The relationship of potential biomarkers with psychological resilience and post-traumatic growth in female patients with breast cancer

**DOI:** 10.1371/journal.pone.0277119

**Published:** 2022-12-16

**Authors:** Ayse Gokcen Gundogmus, Kubra Sezer Katar, Sibel Orsel, Gulfer Ozturk, Kerim Bora Yilmaz

**Affiliations:** 1 Department of Psychiatry, University of Health Sciences, Diskapi Yildirim Beyazit Training and Research Hospital, Ankara, Turkey; 2 Department of Psychiatry, Islahiye State Hospital, Gaziantep, Turkey; 3 Department of Biochemistry, University of Health Sciences, Diskapi Yildirim Beyazit Training and Research Hospital, Ankara, Turkey; 4 Department of Surgery, University of Health Sciences, Gulhane Training and Research Hospital, Ankara, Turkey; 5 Department of Medical and Surgical Research, Institute of Health Sciences, Hacettepe University, Ankara, Turkey; Universita degli Studi Europea di Roma, ITALY

## Abstract

While investigating psychosocial factors on resilience and post-traumatic growth draws attention, research on biological correlates is limited. We investigated the relationship between post-traumatic growth, resilience, post-traumatic stress, and potential biomarkers in female patients with breast cancer (*n* = 71) from the general surgery or oncology clinics. They completed the Post-Traumatic Growth Inventory (PTGI), Connor Davidson Psychological Resilience Scale (CD-RISC), Brief Resilience Scale (BRS), PTSD Checklist for DSM-V, and Hospital Anxiety and Depression Scale. Blood samples were collected for NPY, ALLO, DHEA-S, testosterone, cortisol, and hsCRP levels. The relationship between biochemical parameters and the scales was investigated in the whole patient group and in the subgroup of patients who perceived breast cancer as traumatic. When all the patients were evaluated, hsCRP and depression scores were significantly and positively correlated; and hsCRP, BRS score, and PTGI change in self-perception subscale score were significantly and negatively correlated. There was a significant positive correlation between the ALLO level and the psychological resilience (CD-RISC) score in the patient group who perceived breast cancer as traumatic. It was observed that psychological resilience and PTG were positively correlated, and that multiple biomarkers were associated with psychological resilience in female breast cancer patients. Especially findings regarding ALLO levels and psychological resilience could be a new target for future research.

## Introduction

Breast cancer (BC) is the second most common cancer in the general population and the most common cancer in women [1]. Life-threatening diseases are not always considered traumatic events. However, the diagnosis and treatment of cancer have many features resembling traumatic events [2].

Recently, the possibility of observe a positive outcome from traumatic experiences have received considerable attention [2, 3]. The term post-traumatic growth (PTG) is used to describe the positive outcomes of trauma, and it indicates an improvement in some aspects of the individual’s life (e.g., increased appreciation of life, altered priorities, more meaningful interpersonal relationships, increased sense of personal strength, renewed positivity about the future, existential/spiritual development) [2]. PTG is associated with many psychosocial, sociodemographic, and clinical factors [2, 4]. Contradictory findings were reported regarding the relationship between the perceived level of stress and PTG [2]. While there was no relationship between the level of stress and PTG in some studies [5], others reported that the occurrence of PTG decreases as the perceived level of stress increases [6, 7]. Studies that evaluated stress systematically reported that cancer-related stress was strongly associated with PTG, whereas generalized stress was either not associated with PTG or had a weaker association than cancer-related stress [8, 9].

Another concept that is currently studied in patients with cancer is psychological resilience [10]. It is defined as coping and adapting effectively when faced with loss or adversity and/or recovering when stressors are severe [11]. Studies on patients with BC demonstrated a negative correlation between psychological resilience and depression [10]. The degree of psychological resilience is considered to be a protective factor against psychological distress and depression [10]. In addition, low-level psychological resilience negatively affects cancer mortality in multiple ways, including compromised immune surveillance, accumulated and extended sympathetic and hypothalamic-pituitary-adrenal reactivity to stressors, and the cancer-related mortality rates increases as stress resilience decreases [12].

There is a lack of consensus regarding the relationship between PTG and resilience. Some studies observed that the highest level of PTG occurs when resilience is moderate [13], other studies, noted that high-level resilience is associated with the lowest level of PTG [14], and other research reported that there is a positive relationship between PTG and psychological resilience [15]. Individuals with a high level of resilience are less likely to perceive stressful situations as traumatic and are less likely to experience PTG than those with a low level of resilience [16].

According to the literature, multiple peripheral biomarkers might be related to resilience, including neuropeptide Y (NPY), dehydroepiandrosterone sulfate (DHEA-S), C-reactive protein (CRP), allopregnanolone (ALLO), and testosterone [17, 18]. Many of these biomarkers are also associated with such psychopathologies as post-traumatic stress disorder and major depressive disorder [18–21].

Human and animal studies show that NPY reduces anxiety and stress and may play a role in stress resilience [18, 19, 22, 23]. The role of DHEA-S in the stress response is still unclear, data are indicating that DHEA-S or the DHEA-S/cortisol ratio may be associated with resilience and that they are negatively correlated with the severity of dissociative symptoms [17–21]. However, DHEA-S supplementation has not been observed to affect dissociative symptoms in individuals under stress, and some studies have reported that a high DHEA-S level in individuals with PTSD may be associated with suicidal ideation and the risk of self-harm [24].

Previous studies reported that an elevated CRP level is associated with both depression and anxiety, and that inflammatory processes might play a role in the pathogenesis of these disorders [25]. It was also reported that the CRP level can be considered a biomarker for the risk of developing PTSD [26, 27]. Moreover, a negative correlation was observed between resilience and the CRP level [28]. ALLO, which was clarified to play a role in stress, anxiety, and depression in many studies, acts primarily on GABA_A_ receptors [29] and is considered a potential candidate for treating mood and anxiety disorders [30]. It has a regulatory (homeostasis) effect on the hyperactive hypothalamic-pituitary-adrenocortical (HPA) axis in cases of acute stress [29]. ALLO may play a role in resilience [18, 31].

A negative correlation was observed between a low testosterone level and symptoms of anxiety [32]. Lower testosterone levels were reported in individuals with PTSD than healthy controls [33]. In addition, testosterone was reported to successfully treat depression in men [34, 35]. In contrast to data suggesting that there may be a relationship between testosterone and anxiolysis—and there may be a relationship between testosterone and resilience based on the literature data [19] -some studies report that this inference cannot be yet made [36].

In summary, the experience of illness due to cancer has many similarities with traumatic experiences (i.e., the perception that your life is threatened and the persistence of negative emotions such as fear, dread, and helplessness) [3]. Furthermore, according to the literature psychological resilience plays a role in increasing life expectancy in cancer patients [12]. Although the literature reported a limited number of studies on the relationship between psychological resilience and biomarkers, most studies about this relationship have been conducted in specific populations, such as veterans, military personnel, pregnant women, psychiatry outpatients, and animals. To our knowledge, there are no study in female patients with breast cancer on the relationship between stress-related biomarkers and psychological resilience, and the relationship between stress-related biomarkers and PTG. Findings concerning the relationship between PTG and psychological resilience vary (positive, negative, and curvilinear). The differences in the reported findings might be due to differences in the definition of psychological resilience and the use of different psychological resilience scales. Expanding our understanding of the relationship between PTG and psychological resilience in BC patients, and identifying the biochemical factors associated with resilience and PTG will provide valuable data to clinicians working in this field.

The present study aimed to elucidate the relationship between post-traumatic growth and psychological resilience in female BC patients. Considering the contradictory results in the literature, it was found appropriate to use two different scales for evaluating psychological resilience in different aspects [37–39]. The second aim was to determine if there were any relationships between psychological resilience in female patients with BC and response to trauma (PTG and PTSD), and NPY, testosterone, DHEA-S, cortisol, the DHEA-S/cortisol ratio, ALLO, and the high-sensitivity CRP (hsCRP) levels. Based on the literature it was hypothesized that there would be a positive correlation between both psychological resilience and PTG, and NPY, DHEA-S, the DHEAS/cortisol ratio, testosterone, and ALLO, and a negative correlation between the hsCRP level and resilience and PTG. It is expected that the data obtained in the present study will contribute to the existing literature in this field. Moreover, if a relationship between potential biomarkers and psychological resilience and PTG is observed, it may lead to novel interventions.

## Methods

### Study population and design

This study was conducted at University of Health Sciences, Diskapi Yildirim Beyazit Training and Research Hospital, Department of Psychiatry, Ankara Turkey. The study included 71 literate Turkish female BC patients aged 18–65 years. All female BC patients that presented to the general surgery clinic or oncology clinic for routine follow-up post-breast cancer surgery between January 2019 and January 2020 were informed about the study. Those that agreed to participate and provided written informed consent were included in the study. Following blood collection in the general surgery clinic or oncology clinic, the participants were then referred to the psychiatry outpatient clinic for psychiatric examination and to complete the study forms. Exclusion criteria included epilepsy, dementia, organic mental diseases, such as delirium, mental disability, medical illnesses with significant cognitive sequelae, active alcohol or substance use disorders, use of DHEA-S, testosterone, estrogen, and steroids, diseases that affect the DHEA-S or cortisol levels, such as Cushing’s disease, polycystic ovary syndrome, adrenale adenoma/carcinoma, Crohn’s disease, ulcerative colitis, and hyperthyroidism, and Addison’s disease. In addition, patients diagnosed with obesity and those treated with radioactive material during the previous week were excluded from the present study.

Blood samples were collected between 06.00 and 08.00 a.m., after a minimum of 12 h of fasting, and were immediately centrifuged for 15 min at 1000 ×g. Serum was stored at –80°C in aliquots until analysis. The serum NPY and ALLO levels were measured using a competitive inhibition enzyme immunoassay technique (ELISA), according to the manufacturer’s instructions (Cloud-Clone Corp., Wuhan, PRC). The intra-assay coefficient of variation (CV) for NPY and ALLO was <10%, and the inter-assay CV for NPY and ALLO was <12%. The serum hsCRP level was measured using a Roche Cobas 8000 (702) random access analyzer (Roche Diagnostics). Serum testosterone and cortisol levels were measured using a Roche Cobas 8000 (e801) random access analyzer (Roche Diagnostics). The serum DHEA-S level was measured using an ARCHITECT i2000sr (Abbott Diagnostics).

The study protocol was approved by the University of Health Sciences, Diskapi Yildirim Beyazit Training and Research Hospital Ethics Committee (17.12.2018-57/07). All the patients provided written informed consent, and this study was conducted in accordance with the Declaration of Helsinki.

### Measures

The sociodemographic and clinical evaluation form was used to collect sociodemographic features as age, level of education, and previous psychiatric history. All the patients were administered the Structured Clinical Interview for DSM-5, Clinician Version for diagnostic classification by 2 psychiatrists (AGG and KSK) [40, 41]. The variable “perception of cancer as traumatic" was asked as a yes or no question during the psychiatric evaluation after explaining the concept of traumatic events.

#### Post-Traumatic Growth Inventory (PTGI)

PTGI was developed by Tedeschi and Calhoun (1996) for evaluating psychological development/growth following a traumatic experience [42]. The inventory includes 21 items in five subscales answered using a 6-point Likert-type scale. Higher scores indicate more positive psychological change. The internal consistency coefficient of the original form is α = 0.90. The Turkish version includes three subscales (Changes in self-perception, changes in the philosophy of life, and changes in relationships) [43].

#### Connor-Davidson Psychological Resilience Scale (CD-RISC)

This scale, developed by Connor and Davidson (2003) to measure psychological resilience, consists of 25 items that are answered using a 5-point Likert-type scale. Higher scores are indicative of greater psychological resilience [37, 44]. The Cronbach’s alpha internal consistency coefficient for the original scale is 0.89 [37].

#### The Brief Resilience Scale (BRS)

BRS was developed by Smith et al. (2008) to measure psychological resilience [38]. The scale’s development and the validity-reliability study included four samples, and the internal consistency reliability coefficient is between 0.80 and 0.91 [38]. It is a 6-item 5-point Likert-type scale, and higher BRS scores indicate greater psychological resilience [39].

#### PTSD checklist for DSM-V (PCL-5)

PCL-5 consists of 20 items for assessing the four DSM-5 PTSD symptom clusters (avoidance, re-experiencing, negative alterations, and hyperarousal) [45]. Scale items are answered using a 5-point Likert-type scale ranging from 0 to 4 [46]. Higher scores correspond to greater symptom severity.

#### Hospital Anxiety and Depression Scale (HADS)

HADS was developed to measure anxiety and depression in individuals with physical illnesses [47]. HADS includes two subscales: anxiety and depression. Each of the scale’s 14 items is answered with a 4-point Likert-type scale type ranging from 0 to 3 [48].

### Statistical analysis

Data were analyzed using IBM SPSS Statistics for Windows v.25.0 (IBM Corp., Armonk, NY). Data are presented as number (n) and percentage (%) for categorical (qualitative) variables and as mean (X), standard deviation (SD), and range for numerical (quantitative) variables. Skewness and kurtosis coefficients for the normality of the measurements were determined for the study scale scores; as the assumption of normality was met, the use of parametric analysis methods was considered appropriate. The reliability of the measurements obtained from the study scales was determined based on Cronbach’s alpha values. Pearson’s correlation test was used to determine the relationships between scale scores and biochemical measurements. The t-test for independent groups was used to identify differences in the scale scores and biochemical measurements between the patients who did and did not perceive BC as traumatic. The level of statistical significance was set at *p* < .05.

## Results

Patient demographic and clinical data are given in Table 1. The mean age of the patients was 50.52 ± 9.04, and most of them had undergone modified radical mastectomy, and most of them received radiotherapy (64.8%), chemotherapy (70.4%) and hormone therapy (78.9%). In all, 56.3% of the patients reported that they perceived BC as a traumatic event. Among the patients, 31% had a psychopathology at the time of the study. Descriptive statistics for the biochemical parameters are shown in Table 2.

**Table 1:** Patient demographic and clinical features.

	$\bar{X}\pm \text{SD}$	f (%)
Age, years (range: 31–68)	50.52±9.04	
Time since the diagnosis of cancer, months (range: 2–98)	23.24±18.21	
Age at the time of cancer diagnosis, months (range: 28–66)	48.52±8.97	
Level of Education	Primary school or less	37 (52.11)
	Secondary school	7 (9.86)
	High school	15 (21.13)
	University	12 (16.90)
Surgery	Breast-conserving surgery	16 (22.54)
	Modified Radical Mastectomy	52 (73.24)
	Mastectomy/reconstruction	3 (4.22)
Received radiotherapy		46 (64.79)
Received chemotherapy		50 (70.42)
Received hormone therapy		56 (78.87)
History of psychiatric disease		21 (29.58)
Perceived cancer as traumatic		40 (56.34)
Psychiatric Diagnosis	None	49 (69.01)
	Depressive disorder	12 (16.90)
	Anxiety disorder	4 (5.63)
	Post-traumatic stress disorder	1 (1.41)
	Obsessive-compulsive disorder	2 (2.82)
	Dysthymic disorder	1 (1.41)
	Mixed anxiety and depressive disorder	2 (2.82)

**Table 2:** Descriptive statistics for biochemical parameters.

	Range	Mean ± SD
NPY (pmol/L)	216–1963	786.67±363.24
ALLO (nmol/L)	7–104	47.84±22.62
DHEA-S (microgram/dl)	7–357	121.83±77.80
hsCRP (mg/l)	0–12	3.01±3.15
Cortisol (microgram/dl)	3–39	17.84±7.68
Testosterone (ng/dl)	3–67	20.12±11.46
DHEA-S/Cortisol Ratio	1–25	7.85±5.97

NPY, Neuropeptide Y; ALLO, Allopregnanolone; DHEA-S, Dehydroepiandrosterone sulfate; CRP, C-reactive protein.

The reliability of the measurements obtained from the study scales was determined based on Cronbach’s alpha values, which are given in S1 File. The study scales and sub-scales had acceptable reliability coefficients [49], only 1 of the CD-RISC subscales (the tendency for spirituality) had a low reliability value, because the participants’ responses to the items on this subscale were very similar and exhibited little differentiation. This situation was taken into consideration as the limitation of the research.

In total, 40 of the patients perceived BC as traumatic, versus 31 who did not perceive it as traumatic. There were no significant differences in NPY, ALLO, DHEA-S, hsCRP, cortisol, testosterone, the DHEA-S/cortisol ratio, the level of anxiety and depression, or PTGI scores between the patients who did and did not perceive cancer as traumatic (*p* > 0.05). There was a significant difference between the patients who did and did not perceive cancer as traumatic concerning the BRS score, CD-RISC tenacity and personal competence, and tolerance of negative affect subscale scores, or CD-RISC total score (*p* < 0.05). Patients who did not perceive cancer as traumatic had higher BRS and CD-RISC total scores, CD-RISC tenacity and personal competence, and tolerance of negative affect subscale scores, whereas those who perceived cancer as traumatic had higher PCL-5 total scores and PCL-5 negative alterations subscale scores. There wasn’t a significant difference in the PTGI score between the patients who did and did not perceive BC as traumatic, whereas there were significant differences in the other scale scores; therefore, the relationship between PTGI and these other scales was analyzed after grouping the patients according to their perception of BC as traumatic or not. In both groups there was a positive correlation between the CD-RISC and PTGI total scores, and most of their subscales’ scores (*p* < 0.05). On the other hand, while there was a relationship with BRS and PTGI total score in the patient group who perceive cancer as traumatic, no relationship was found for the other group. Lastly, there was a negative correlation between PTGI and depression scores in patients who do not perceive cancer as traumatic. Relationships between scales and subscales’ scores and PTGI are shown in Table 3.

**Table 3:** The relationship between PTGI scores and other scale scores (for two groups).

	Patients who do not perceive the disease as traumatic (n = 31)				Patients who perceive the disease as traumatic (n = 40)			
Scales and subscales	1	2	3	4	1	2	3	4
HAD-anxiety	-.176	-.115	-.213	-.182	-.076	-.246	.004	-.113
HAD-depression	-.372*	-.324	-.361*	-.382*	-.236	-.260	-.105	-.237
BRS	.141	.125	.049	.120	.373*	.308	.105	.325*
CD-RISC								
-Tenacity and Personal Competence	.437**	.320	.468**	.444**	.542**	.416**	.386*	.535**
-Tolerance of Negative Affect	.491**	.413**	.464**	.497**	.414**	.332*	.310	.418**
-Tendency toward Spirituality	.359*	.506**	.536**	.479**	-.029	-.125	.035	-.042
-Total	.516**	.441**	.564**	.546**	.477**	.353*	.354*	.471**
PCL-5								
-Re-Experiencing	-.170	-.084	-.211	-.169	.026	-.042	.117	.037
-Avoidance	-.009	.046	-.073	-.012	.203	.036	.076	.143
-Negative alterations	-.191	-.132	-.232	-.200	.085	-.072	.130	.065
- Hyperarousal	.027	.083	-.109	.007	.043	-.002	.012	.026
-Total	-.110	-.036	-.195	-.121	.082	-.036	.097	.063

*p < .05,

**p < .01,

Pearson’s correlation. 1 = PTGI Changes in self-perception, 2 = PTGI Changes in the philosophy of life, 3 = PTGI Changes in relationship, 4 = PTGI Total, HAD, Hospital Anxiety and Depression Scale; BRS, Brief Resilience Scale; CD-RISC, Connor-Davidson Psychological Resilience Scale; PCL-5, PTSD Checklist for DSM-V; PTGI, Post-traumatic Growth Inventory.

The relationship between possible biomarkers (NPY, ALLO, DHEA-S, hsCRP, cortisol, testosterone, the DHEA-S/cortisol ratio), and depression, anxiety, BRS, CD-RISC, PCL-5, and PTGI are presented in Table 4.

**Table 4:** The relationship between possible biomarkers and study scale scores.

	Entire Patient Group (n = 71)							Patients that perceive the disease as traumatic (n = 40)						
	1	2	3	4	5	6	7	1	2	3	4	5	6	7
HAD-anxiety	-.100	.084	.209	.194	.199	.126	.101	.156	-.124	.170	.050	.277	.038	.029
HAD-depression	-.067	-.073	-.030	.287*	.086	-.076	-.074	.028	-.170	-.149	.105	.145	-.190	-.143
BRS	.013	-.070	-.040	-.269*	-.093	-.049	.040	-.039	.133	-.078	-.212	-.202	-.039	.109
CD-RISC														
-Tenacity and Personal Competence	.066	.129	.202	-.088	-.150	-.014	.230	-.057	.317*	.215	-.172	-.235	.126	.268
-Tolerance of Negative Affect	.112	.124	.129	-.121	-.061	-.039	.115	.015	.309	.202	-.059	-.155	.073	.196
-Tendency toward Spirituality	-.032	.216	.154	-.118	-.194	-.062	.205	-.134	.154	.113	-.237	-.312	-.001	.177
-Total	.074	.161	.200	-.117	-.151	-.032	.222	-.055	.329*	.222	-.170	-.253	.104	.265
PCL-5														
-Re-Experiencing	-.153	.099	.228	.181	.156	.082	.077	-.031	-.040	.261	.029	.258	.054	.053
-Avoidance	-.079	.155	.173	.018	.198	.013	.081	-.015	.078	.167	-.239	.192	-.122	.056
-Negative alterations	-.228	.097	.054	.118	.102	-.060	-.053	-.090	-.034	-.061	-.024	.116	-.150	-.144
- Hyperarousal	-.174	.013	.044	.064	.064	-.141	.051	-.025	-.044	-.013	-.007	-.028	-.270	.105
-Total	-.201	.089	.119	.119	.129	-.049	.028	-.055	-.029	.062	-.035	.130	-.152	-.001
PTGI														
-Changes in self-perception	.002	.145	.184	-.255*	.064	.089	.181	-.047	.265	.165	-.287	.078	.080	.167
-Changes in the philosophy of life	-.100	.106	.208	-.194	.113	.056	.142	-.202	.139	.157	-.220	.144	.057	.104
-Changes in relationship	.041	-.046	.044	-.089	.068	-.065	-.014	.001	-.038	-.068	-.090	.172	-.105	-.199
-Total	-.016	.092	.169	-.215	.087	.043	.131	-.086	.172	.115	-.248	.139	.028	.059

*p < 0.05,

**p < 0.01,

Pearson’s correlation. 1, Neuropeptide Y; 2, Allopregnanolone; 3, Dehydroepiandrosterone sulfate; 4, C-reactive protein; 5, Cortisol; 6, Testosterone; 7, DHEA-S/Cortisol; HAD, Hospital Anxiety and Depression Scale; BRS, Brief Resilience Scale; CD-RISC, Connor-Davidson Psychological Resilience Scale; PCL-5, PTSD Checklist for DSM-V; PTGI, Post-Traumatic Growth Inventory.

When all the patients were evaluated, there was a significant positive correlation between hsCRP and the HAD-depression score, and a significant negative correlation between hsCRP and the BRS score and PTGI change in self-perception subscale score. In patients who perceived BC as traumatic, there was a significant positive correlation between ALLO and the CD-RISC tenacity and personal competence subscale score, and CD-RISC total score (*p* < 0.05).

## Discussion

The present study investigated the relationship between PTG and psychological resilience in female patients with BC. The present study also examined the relationship between possible biomarkers, PTG, psychological resilience, and symptoms of post-traumatic stress, according to the entire patient group and in a subgroup of patients who perceived cancer as traumatic.

In the present study, the patients with greater psychological resilience, evaluated with CD-RISC, had a greater degree of PTG. On the other hand, when resilience was evaluated with BRS, we found that it was positively associated with PTG only in patients who perceived BC as traumatic. Many scales examine psychological resilience in the literature, and these scales evaluate different dimensions of psychological resilience. Both the BRS and CD-RISC assess resilience; however, BRS focuses on post-traumatic resilience (recovery), whereas CD-RISC evaluates general psychological resilience (rather than recovery and returning to a former functional state, it focuses on factors affecting psychological resilience, such as self-efficacy, a sense of humor, patience, optimism, and faith) [12, 50]. Findings regarding the correlation between psychological resilience and PTG vary [16], and in the present study, based on the CD-RISC and BRS the relationship between PTG and psychological resilience also varied—according to CD-RISC there was a positive relationship between PTG and psychological resilience in both groups, but according to BRS this relationship was only observed in the patients who perceived BC as traumatic [16]. The observed differences based on CD-RISC and BRS might have been due to the fact that the 2 scales measure different aspects of resilience. The present findings indicate that the ability to recover from trauma rapidly is associated with PTG only in patients who perceive BC as traumatic; therefore, such patients should be specifically targeted with interventions to increase resilience in terms of rapid recovery. Based on the present findings, we think that the characteristics that constitute general psychological resilience are associated with PTG independently of perceiving of events as traumatic [18].

Although traumatic stress symptoms are frequently observed in patients with BC, the relationship between post-traumatic stress symptoms and PTG is complex, and there is the possibility that these are independent constructs [2, 51]. The relationship between PTSD and PTG may even vary according to the type of trauma. For example, in the case of severe illness (in self or significant other), the relationship between PTG and PTSD is weak or nonexistent [52]. In the present study, there was no relationship between PTG and symptoms of post-traumatic stress. The interaction between these phenomena should be investigated in detail [53].

A notable finding of the present study is that there was a negative relationship between depression and PTG in patients who did not perceive BC as traumatic, that is, in patients with a high level of psychological resilience. Also, there was no significant relationship between depression and PTG in patients who perceive BC as traumatic. Most relevant studies report that there is negative relationship between depressive symptoms and PTG [54].

In the present study, 56.3% of the patients with BC perceived cancer as traumatic and perception of cancer as a traumatic stressor was associated with greater levels of PTG in the literature [55]. On the other hand, there are contradictory findings regarding the relationship between the perceived level of stress and PTG in the literature, and cancer-related stress was strongly associated with PTG [3, 8, 9]. When the participants were grouped according to those who did and did not perceive cancer as traumatic, there were not any differences in blood levels of the studied biomarkers, anxiety and depression levels or PTG scores, and as expected, those who did not perceive cancer as traumatic had higher psychological resilience. In contrast, the patients who perceived cancer as traumatic had more symptoms of post-traumatic stress. The findings obtained in the present study are in agreement with findings obtained in the previous studies that suggest that psychological resilience can protect against traumatization [22, 42, 56]. In the present study there weren’t any differences in the studied biomarkers between the two groups, which might have been due to the small study population. Better sampling criteria, diversifying patients according to disease staging, and more detailed evaluation of the perception of BC as traumatic or not might have yielded more robust biomarker findings.

Many studies indicate a relationship between hsCRP and depression [19]. Some studies noted a relationship between anxiety symptoms and the CRP level [20]. There are many studies on the relationship between the CRP level and PTSD, and it has been asserted that CRP can be a biomarker of the risk of PTSD [26, 27]. There was a positive correlation between the hsCRP level and the HAD-depression score among all the patients, which is consistent with the literature [26, 27, 57]. On the other hand, this correlation did not exist in the group of patients who perceived BC as a traumatic event, which might have been due to the lack of addressing some unknown factors. According to the literature, hsCRP may not be a suitable biomarker of psychological distress due to the existence of many confounding effects [58]. We think larger-scale studies are needed for additional clarification.

Similar to studies that reported a negative correlation between resilience and the CRP level [28], in the present study, there was a negative correlation between hsCRP and post-traumatic psychological resilience based on the BRS score in all the included patients. The present study’s patients with a high hsCRP level took more time to return to their pre-trauma level of functioning than those with a low hsCRP level might have been due to the negative effects of depressive symptoms, which should be further studied.

Again, there was a negative correlation between the hsCRP level and the PTGI change in self-perception subscale score in patients in the present study. In the original form of PTGI, the change in self-perception subscale was evaluated as self-confidence—knowing that challenges can be overcome, accepting life as it is, and discovering that one is more potent than he/she thinks [42]. In the Turkish adaptation study, this subscale included many more dimensions [43]. Previous studies reported that the most frequently reported positive change in patients with cancer is that they feel psychologically more robust and more confident [59]. To our knowledge, no study has examined the relationship between hsCRP and PTG.

Among the patients in the present study who perceived cancer as traumatic, the level of psychological resilience (CD-RISC) was higher in those with higher levels of ALLO, which is in line with findings suggesting that ALLO might play a role in resilience [18, 31]. Findings regarding the ALLO level in cases of acute stress in humans are inconsistent, and it is hypothesized that this may be due to the biphasic effects of ALLO (anxiogenic in lower doses and anxiolytic in higher doses) and that a low ALLO level in cases of chronic exposure to stress may be associated with a compensatory mechanism related to hypersensitization of GABA_A_ receptors [29]. Low ALLO levels have been associated with major depression, anxiety disorders, and premenstrual dysphoric disorder [33]. The high frequency of postpartum depression in women with a low ALLO level and recommendation of its use to treat postpartum depression supports these findings [60, 61]. Although studies in the literature also suggest that severity of stress intensity and different nature of stressors may affect ALLO levels [29]. In the present study, data were evaluated in accordance with previous studies, as a high ALLO level was associated with a higher degree of psychological resilience, and psychological resilience was associated with fewer anxiety and depression symptoms.

Some studies have reported a relationship between the DHEA-S/cortisol ratio and psychological resilience [18, 19]. It was also highlighted in previous studies that individuals with a high DHEA-S/cortisol ratio have fewer dissociative symptoms under stress and have better behavioral performance [21]. These findings support the finding of a high level of psychological resilience in individuals with a high DHEA-S/cortisol ratio, considering the inverse relationship between psychological resilience and psychopathology [10]. Drawing on the literature, DHEA-S is likely to be considered a potential biomarker of resilience. However, its common effects (modulation of cortisol production, precursor of anabolic steroids, stress and pro-inflammatory cytokines, and modulation of multiple neurotransmitter systems, such as the HPA axis) may limit its ability to be considered a specific biomarker of resilience [22]. The abovementioned effect might be why in the present study, there was not a relationship between the DHEA-S/cortisol ratio and any of our study variables.

In the present study, there was no relationship between testosterone and any other variables of the study, and given that most earlier studies were conducted on men, these findings might be related to gender [32, 62]. On the other hand, data suggest that there may be a relationship between testosterone and anxiolysis in women and that there may be a relationship between testosterone and resilience.

The present study has limitations, including a small patient population and a single-center design, making it challenging to generalize the findings. As there were differences in the time since diagnosis between the patients, the observed relationship between PTG and the studied biomarkers cannot be generalized even though statistically no effect of it has been found on study scales’ scores except hyper-arousal subscale of PCL-5. The present correlational study’s findings can be used to simply identify relationships, but cannot demonstrate the causality of the relationships. The reliance on patient self-reports concerning inflammatory diseases and drugs that can affect the studied biomarker can be considered a limitation. In addition, some of the patients included in the study had psychopathology, which may have affected the findings. Another limitation is that the patients were asked only a single question about whether BC was perceived as traumatic or not, and their perceptions of the BC experience being traumatic or not was not evaluated during the acute period. Moreover, the patient group only included BC patients that primarily underwent mastectomy, chemotherapy, and radiotherapy (advanced stage patients); therefore, the findings cannot be generalized to all female BC patients.

## Conclusion

Our findings in this study showed a positive correlation between psychological resilience and PTG, while many previous studies reported contradictory results. Furthermore, the relationship between biochemical parameters and PTG, psychological resilience, and post-traumatic stress was investigated in the entire BC patient group and in a subgroup of the patients who perceived BC as traumatic. In the present study the observed correlations between the studied biomarkers, and PTG and psychological resilience varied. Earlier studies on these biomarkers focused to a greater degree on their relationships with psychopathology [29, 33]; their relationships with psychological resilience has been investigated only recently. To the best of our knowledge the present study is the first to examine the relationship between PTG and the included biomarkers. Additionally, the observed positive relationship between ALLO and psychological resilience effectively expands the relevant literature. Given that it was reported that psychological resilience increases life expectancy in patients with cancer, it is crucial to investigate the associated biochemical and psychological factors. While trying to ensure the patient’s survival with BC, evaluating the psychological effects of the disease and understanding the psychological changes (such as traumatization and post-traumatic growth) experienced by the patients due to BC will facilitate optimal disease management by clinicians.

## Supporting information

S1 File Reliability of the measurements (R). This is the “reliability values of the measurements obtained from the measurement tools”.
(DOCX)

S2 File SPSS GG. This is the study’s underlying data set.
(SAV)
