# Supplementary material for: The relationship of potential biomarkers with psychological resilience and post-traumatic growth in female patients with breast cancer
Source: PLoS One. 2022 Dec 16;17(12):e0277119. doi: 10.1371/journal.pone.0277119 (PMC9757578; doi:10.1371/journal.pone.0277119)
Supplement: S1 File — This is the “reliability values of the measurements obtained from the measurement tools”. (DOCX) [file pone.0277119.s001.docx]

In order to provide evidence for the reliability of the measurements obtained from the measurement tools within the scope of the research, Cronbach's alpha values were calculated and given in Table 1.

**Table 1. Reliability values of the measurements obtained from the measurement tools**

| Measurement tools and subscales | Number of items | Cronbach α |
| --- | --- | --- |
| Brief Psychological Resilience Scale | 6 | .748 |
| HAD Anxiety | 7 | .844 |
| HAD Depression | 7 | .750 |
| PCL-5 Re-experiencing | 5 | .841 |
| PCL-5 Avoidance | 2 | .897 |
| PCL-5 Negative alterations | 7 | .889 |
| PCL-5 Hyper-arousal | 6 | .858 |
| PCL-5 Total Score | 20 | .944 |
| CD-RISC Tenacity and Personal Competence | 15 | .901 |
| CD-RISC Tolerance of Negative Affect | 6 | .817 |
| CD-RISC Tendency toward Spirituality | 4 | .403 |
| CD-RISC Total Score | 25 | .916 |
| PTGI Changes in self-perception | 10 | .901 |
| PTGI Changes in the philosophy of life | 6 | .797 |
| PTGI Changes in relationship | 5 | .831 |
| PTGI Total Score | 21 | .936 |

HAD, Hospital Anxiety and Depression Scale; CD-RISC, Connor-Davidson Psychological Resilience Scale; PCL-5, PTSD Checklist for DSM-V; PTGI, Post-Traumatic Growth Inventory

When Table 1 is examined, it is seen that the scales and sub-dimensions used in the research have acceptable reliability coefficients (Charter, 2003). It is seen that only one of the sub-dimensions of the CD-RISC scale, the tendency toward spirituality has a low reliability value. One of the reasons for this situation is that the responses of the participants to the items in this sub-dimension are very close to each other and the differentiation is very small. This situation was taken into consideration as the limitation of the research.

**References**

Charter, R. A. (2003). A breakdown of reliability coefficients by test type and reliability method, and the clinical implications of low reliability. *The Journal of general psychology*, *130*(3), 290-304. [https://doi.org/10.1080/00221300309601160](about:blank)
